# Waist circumference and insulin resistance in elderly men: an analysis of Kahrizak elderly study

**DOI:** 10.1186/2251-6581-13-28

**Published:** 2014-02-04

**Authors:** Mojdeh Mirarefin, Farshad Sharifi, Hossein Fakhrzadeh, Mohammad Reza Amini, Maryam Ghaderpanahi, Nahid Zerafati Shoa, Zohreh Badamchizadeh, Yaser TajalizadeKhoob, Neda Nazari, Bagher Larijani

**Affiliations:** 1 Elderly Health Research Center, Endocrinology and Metabolism Population Sciences Institute, Tehran University of Medical Sciences, Tehran, Iran; 2 Endocrinology and Metabolism Research Institute, Tehran University of Medical Sciences, Tehran, Iran

**Keywords:** Waist circumference, Insulin resistance, Metabolic syndrome, Elderly

## Abstract

**Background:**

Diagnosis of the metabolic syndrome (MS) is crucial for health care practitioners to identify at risk people for early treatment. Visceral obesity may make unnecessary other laborious measures of insulin resistance. The aim of this study was to see whether waist circumference (WC) can predict insulin resistance as well as MS in a group of Iranian elderly.

**Methods:**

Out of 94 nondiabetic elderly, thirty three subjects were recognized with MS. MS diagnosis was based on NCEP ATP III (National Cholesterol Education Program Adult Treatment Panel III) and IDF (International Diabetes Federation) definitions. HOMA (Homeostasis Model Assessment) index was used to measure insulin sensitivity. Insulin resistance (IR) was defined as top quartile of HOMA.

**Results:**

In both sexes, WC and HOMA index were significantly positively correlated. The optimal waist circumference (OWC) cutoff point was 94.5 cm for men and 90.5 cm for women. The high sensitivity (0.80) and specificity (0.84) of WC in males indicates the proportion of IR which is correctly identified and recognizes all non-IR males as such. In regression model only the TG level was associated with WC. But the WC is strongly associated with HOMA-IR.

**Conclusions:**

While OWC is very likely a good measure to exclude non-IR subjects in our study, determination of optimal WC to identify elderly IR subjects warrants further study in a larger sample of the general population.

## Introduction

The clustering of a group of heart disease risk factors, and their association with insulin resistance, led investigators to propose the existence of the condition “metabolic syndrome (MetS)” [1] or “insulin resistance” [2]. This health condition is very prevalent in Iranian population [3] and its frequency is higher among older Iranian people [3]. Diagnosis of the metabolic syndrome is crucial for health care practitioners to identify at risk people for early treatment, long-term management and cardiovascular disease prevention. Based on a definition published by the International Diabetes Federation (IDF) [4] and National Cholesterol Education Program Adult Treatment Panel III [5] (NCEP-ATP III) [5], abdominal obesity is a key factor for diagnosis of metabolic syndrome and it probably is the potential link between metabolic syndrome and insulin resistance [6]. On the other hand waist circumference (WC) is an independent risk factor for cardiovascular disease [1]. Blood sampling is required for measurement of the other components of metabolic syndrome. As well as WC is an easy and clinically useful scale to identify metabolic syndrome. It has been suggested that in elderly men WC is a better indicator of metabolic abnormalities than percent body fat [7]. It is also a good measure of central adiposity [8-10]. Intra-abdominal obesity or visceral fat is strongly associated with metabolic disturbances and insulin resistance [11,12]. Insulin sensitivity is traditionally determined by euglycaemic-hyperinsulinaemic clamp technique [13], but in general population; it is more convenient and cost-effective to estimate homoeostasis model assessment (HOMA index) using plasma glucose and insulin [14,15].

The cut-point of WC among older people is not clear. Some longitudinal studies recommended higher cut-point for the elderly than the adult population [16]. While it has been stated that WC < 100 excludes insulin resistance in both sexes in adults, however in elderly women a WC >88 cm has been shown to indicate a high likelihood of insulin resistance and is almost as good as MetS defined using the NCEP criteria in predicting Insulin Resistance (IR) [17]. Due to ethnic-specific values for WC in IDF definition as well as its determination in an Iranian population [18], it is noteworthy to see whether waist circumference would predict insulin resistance as good as metabolic syndrome in the Iranian elderly people.

## Methods

A cross-sectional study was conducted among elderly residents of the Kahrizak Charity Foundation between 2007–2008 in Tehran, Iran. Subjects ≥ 60 years of age were considered elderly. Data collection was performed during annual health assessment of elderly subjects. Among identified living residents of Kahrizak, volunteers were recruited as prospective participants. Primary data such as age, sex, cause of admission, code of each resident, residency duration and admission unit were exploited from medical records and recorded in a specific questionnaire designed for the purpose of study.

### Subjects and study criteria

Data for this study were from the baseline data of Kahrizak Elderly cohort study; this was a longitudinal institutionalized based study for recognizing of risk factors of the morbidity and mortality in older people which was conducted in the Kahrizak Charity Foundation. Additional detail information about this study was explained elsewhere [19]. Those aged ≥ 60 who were not bedridden, considered healthy, according to the Mini Mental State Examination (MMSE) questionnaire (score >21) [20], and were non-diabetic , had no end stage disease such as cancer, chronic kidney disease or liver failure and volunteer to participate included in the study.

Weight was measured in light clothing with bare feet to the nearest 0.1 Kg using electronic scale and Height was measured without shoes as the distance from the top of the head to the bottom of the feet to the nearest 0.1 cm using a stadiometer. Waist measurement was taken from the midpoint between the iliac crest and the lower ribs measured at the sides while standing. Blood pressure was measured twice by a trained team according to The Seventh Report of the Joint National Committee on Prevention, Detection, Evaluation, and Treatment of High Blood Pressure (JNC VII) criteria. Briefly, it was measured with an automatic sphygmomanometer (Omron M7, Japan) in the right arm in the sitting position after resting for 5 minutes. Participants were recommended to avoid alcohol, cigarette smoking, caffeinated beverages, and exercise for at least 30 min before their blood pressure measurement. An average of the two measurements was recorded with 3 days interval during a week. Calibration with a mercury sphygmomanometer had been done after every 100 measurements (The Seventh Report of the Joint National) [21]. Diabetes was defined according to the American Diabetes Association [22].

The study protocol was approved by Endocrinology and Metabolism Institute ethics committee and conformed to the Declaration of Helsinki. All subjects gave written informed consent.

### Biochemical analysis

Fasting venous samples were obtained. Blood samples were centrifuged (10 min, RT, at 2000 RPM) in room temperature. Serum aliquots were divided into micro tubes and stored at -32°C until measurement. Fasting blood sugar (FBS), Triglyceride (TG), Total Cholesterol (TC), high density lipoprotein Cholesterol (HDL–C) was measured by enzymatic method (Pars Azmun, Iran). Plasma insulin was determined using ELISA method (Denmark, Monobind, 2008).

### Diagnosis

According to modified ATP (Adult Treatment Panel) III criteria metabolic syndrome is identified by the presence of at least three of the following components: increased WC (>102 cm for men, > 88 cm for women), blood pressure elevation (≥ 130/85 mmHg) and/or use of anti-hypertensive medications, low HDL-C (<40 mg/dl in men, < 50 mg/dl in women), high TG (≥ 150 mg/dl), hyperglycemia (fasting glucose ≥ 100 mg/dl) and/or anti-diabetic medications [5]. Metabolic syndrome based on the IDF definition was defined as the ethnic definition of WC for Iranian population (≥ 91.5 cm for men, ≥ 85.5 cm for women) [19] plus any of these two: blood pressure elevation, reduced HDL cholesterol, raised TG (with the same cut-offs as ATP III), or raised fasting plasma glucose ( ≥ 100 mg/dl ).

HOMA index was calculated as described by Matthews [fasting plasma glucose (mmol/L) × fasting insulin (μU/ml)/22.5] [14].

According to European Group for the Study of Insulin Resistance [23], IR was defined as top quartile of HOMA in non-diabetic subjects.

### Statistical analysis

Statistical analysis was performed using SPSS software, 15.0 (SPSS Inc Chicago). Values less than 0.05 were considered significant. Data were stratified by sex. Normality of values was checked by Kolmogorov- Smirnov test. To normalize skewed values, Log_10_ transformation was used for Triglyceride and HOMA-IR. The association between WC and components of metabolic syndrome was undertaken using Pearson correlation. We used Receiver Operating Characteristic (ROC) curve to determine the cutoff point for WC in predicting IR. For determining the accuracy of the ROC curve, Area under the ROC Curve (AURC) was estimated for all components of metabolic syndrome. To confirm the results of the ROC curve, linear regression model was performed.

## Results

One-hundred and forty five subjects ≥ 60 years participated in this study. Fifty one subjects were excluded because they had diabetes. 94 subjects (42 men and 52 women) were included in the study (Table 1). Then HOMA-IR and TG no had normal distribution in the Kolmogorov- Smirnov test. According to NCEP and IDF criteria, the prevalence of metabolic syndrome in the first 145 participants was 37.6% (23.2% male & 48.1% female) and 36.8% (26.8% male & 44.2% female), respectively. After excluding diabetic ones, the prevalence of metabolic syndrome based on the definition of NCEP and IDF became 29.7% (13% male & 41.5% female) and 27% (13% male & 36% female), respectively.

**Table 1:** General characteristic of participants

**Variable**	**Men (n = 42)**	**Women (n = 52)**
	**Mean ± SD**	**Mean ± SD**
Waist circumference (cm)	87.94 ± 11.48	89.91 ± 12.78
Body weight (kg)	62.18 ± 13.90	56.19 ± 13.86
Body mass index (kg/m^2^)	23.16 ± 4.42	25.31 ± 5.78
Triglycerides (mg/dl)	110.29 ± 56.25	152.17 ± 72.88
HDL-cholesterol (mg/dl)	43.18 ±13.01	46.53 ± 13.15
LDL-cholesterol (mg/dl)	106.56 ± 25.81	116.46 ± 30.41
Systolic blood pressure (mmHg)	136.91 ± 27.05	126.68 ± 23.57
Diastolic blood pressure (mmHg)	75.97 ±14.34	73.59 ± 13.67
Plasma glucose (mmol/L)	5.57 ± 0.67	5.41 ± 0.82
Serum insulin (mU/L)	4.76 ± 4.83	6.15 ± 5.19
HOMA index	1.21 ± 1.24	1.58 ± 1.64

**Figure 1:**
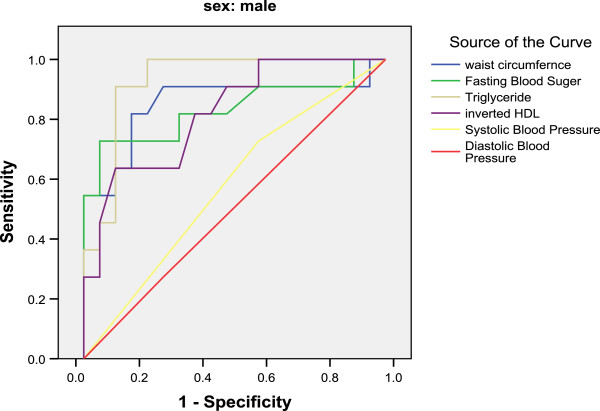
ROC curves of the components of metabolic syndrome on criterion of insulin resistance for men.

**Figure 2:**
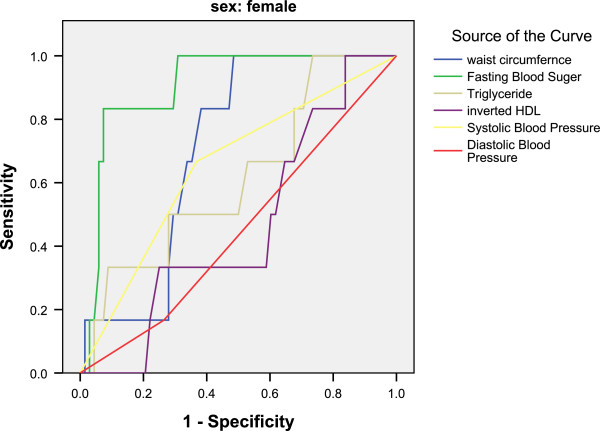
ROC curves of the components of metabolic syndrome on criterion of insulin resistance for women.

The ROC curve analysis was conducted for the components of metabolic syndrome on the criterion of HOMA-IR which was categorized based on first quartile and three other quartiles. The Figures 1 and 2 were demonstrated this ROC curve analysis in male and female.

WC and HOMA index were significantly positively correlated in men (correlation coefficient: 0.590, P < 0.001) and women (correlation coefficient: 0.246, P < 0.05). The AURC _TG_ was greater than that of the other components of metabolic syndrome and the AURC _WC_ ranked second. In women the AURC _FBS_ was greater than that of the other components of metabolic syndrome and again the AURC _WC_ ranked second. AURC for WC was 0.83 (95% CI: 0.68-0.99) in men and 0.70 (95% CI: 0.56-0.85) in women (Table 2).

**Table 2:** Area under the curve of MS components according to sex

**Measure**	**Men**		**Women**	
	**AURC**	**P**_**value**_	**AURC**	**P**_**value**_
Waist Circumference	0.83	0.01	0.70	0.04
FBS	0.76	0.02	0.85	0.01
TG	0.87	0.00	0.53	0.72
HDL	0.77	0.06	0.44	0.90
SBP	0.54	0.42	0.54	0.45
DBP	0.50	0.52	0.43	0.71

*FBS*; Fasting Blood Sugar, *TG*; Triglyceride, *HDL*; High Density Lipoprotein, *SBP*; Systolic Blood Pressure, *DBP*; Diastolic Blood Pressure.

The optimal cutoff point with the highest sensitivity and specificity derived from the ROC curve in our study was 94.5 cm for men and 90.5 cm for women. Sensitivity, specificity, positive predictive value (PPV), negative predictive value (NPV), and proportion correctly classified (PCC) for IR (Table 3). In men sensitivity, specificity and NPV of OWC for IR was higher than those of NCEP_WC_ and IDF_WC_. However, in women’s sensitivity, specificity and NPV of IDF_WC_ for IR was greater than those of OWC and NCEP_WC_. In the end PCC of IDF_WC_ was greater than PCC of NCEP_WC_ and OWC.

**Table 3:** Predictive value of insulin resistance using optimal waist circumference, NCEP and IDF definition of MS

**Measure**	**Men**			**Women**		
	**NCEP**	**IDF**	**WC**	**NCEP**	**IDF**	**WC**
Sensitivity	0.75	0.77	0.80	0.48	0.59	0.50
Specificity	0.79	0.83	0.84	0.57	0.63	0.52
Positive predictive value	0.33	0.6	0.4	0.40	0.23	0.18
Negative predictive value	0.91	0.94	0.96	0.55	0.85	0.82
*Proportion correctly classified*	0.83	0.9	0.83	0.52	0.61	0.51

In univariable and multivariable logistic regression, there was only an association between WC and TG between the components of MetS. But there was a strong association between WC and HOMA index (Table 4).

**Table 4:** Association between the components of metabolic syndrome and insulin resistance with OWC in univariate and multivariable logistic regression models

**Variables**	**Univariate analysis**	Multivariable analysis*
	**OR (CI 95% OR)**	**OR (CI 95% OR)**
High blood pressure	0.41 (0.17 – 1.02)	0.41 (0.17 – 1.02)
High blood triglyceride levels	2.86 (1.115 – 7.08)	2.88 (1.16 – 7.15)
Low blood HDL-C levels	2.09 (0.83 – 5.26)	2.10 (0.83 – 5.28)
High blood glucose levels	2.42 (0.46 – 12.80)	2.69 (0.49 – 14.73)
High HOMA-IR index	6.00 (1.62 – 22.18)	6.12 (1.64 – 22.80)

Notice: The cut-points for construction of WC dummy variable were 90.5 cm for women and 94.5 for men.

*Adjusted for age.

## Discussion

In this study, we compared OWC and NCEP _WC_ as well as IDF _WC_ in the prediction of IR with non-diabetic Iranian elderly residents of the Kahrizak Charity Foundation. We found OWC is a better predictor of IR after TG in men and after FBS in women. In men NPV of OWC was greater than NPV’s of IDF _WC_ and NCEP_WC._ This means that with a probability of 0.96 a man with WC less than 94.5 will not have IR. In women, on the other hand, we can claim that with a probability of 0.82 a female with WC less than 90.5 will not be affected by IR. While OWC is very likely a good measure to exclude non-IR subjects; IDF_WC_ has a better predictive value for IR than NCEP_WC_ and OWC in this population of elderly Iranians. In clinical practice because measurement of WC is easier than TG, it is justified to investigate its applicability to predict IR. Although AURC_TG_ in men and AURC_FBS_ in women were greater than AURC_WC_, however OWC was good (0.8) in men and fair (0.7) in women at separating non-IR from IR subjects.

There is some pathophysiologic justification about waist circumference and insulin resistance. The waist circumference is one of the most user index for abdominal obesity and visceral fat accumulation. The studies have been shown that accumulation of adipose tissue could increase releasing of free fatty acids, inflammatory cytokines and decrease secretion of adiponectin. These changes in mediators could decrease insulin sensitivity in muscle tissues and subsequently reduce insulin-mediated glucose uptake [24].

Moreover, OWC as determined by the ROC curve indicates the point with the highest sensitivity and specificity. The high sensitivity of OWC (0.80) among the male elderly indicates the proportion of IR which was correctly diagnosed and its high specificity (0.84) means the proportion of correctly identified non-IR. Waist circumference is a central measure to describe the metabolic syndrome as defined by NCEP: ATP-III and IDF definition [4]. In addition, metabolic syndrome and insulin resistance are strongly associated with intra-abdominal obesity or visceral fat [11,12]. In 2005 German investigators performed a nationwide screening of 35 869 unselected patients to estimate their cardiovascular risk. At the end, they concluded that routine measurement of WC in primary care attendees is a suitable screening tool to identify those with high cardiovascular risk in which a further diagnostic work-up is justified [25]. Moreover, a recent population-based study has shown the suitability of WC in identifying cardiometabolic conditions and risk factors in primary care facilities. They have investigated whether WC or BMI were strong associates of CVD, diabetes mellitus, lipid disorders and hypertension. The results of such studies have shown WC as a strong associate of CVD and diabetes mellitus even in those lean or overweight by BMI. The authors attributed such effect to the abdominal fat accumulation which contributes to the insulin resistance [26]. Another study in a large population shown that waist circumference could use as a single anthropometric predictor of all cause of mortality [27]. A study in Iran was shown that the WC is better than the BMI and waist to hip ratio could predict the metabolic syndrome [28]. Another study reported that the WC more related to metabolic syndrome in the population who aged ≥ 50 years than those aged < 50 years [29]. A study reported that the optimal BMI and WC should redefine in older people and may be the cut-point for the categorizing of these anthropometric measures about this age group should consider larger than the adult population [16].

On the other hand the correlation of abdominal obesity with insulin resistance is to the extent that more laborious measures of insulin resistance seem unnecessary [30]. In the elderly (67–78 y) WC and abdominal sagittal diameter are reported to be more closely correlated with to metabolic risk factors [31].

The WC cutoff point has been determined for the diagnosis of metabolic syndrome in Iranian adult population [19]. In this study, we assessed the suitability of WC in predicting IR in elderly Iranians. While the current study has been shown the suitability of OWC in identifying IR elderly men, Nilsson et al. [18] reported waist circumference as a useful tool in predicting IR in Swedish elderly women (WC >88 indicated a high likelihood of IR). Discrepancies between our results and Nilsson et al. [18] may be due to difference in identifying MS subjects using IDF criteria. We used definition of WC for Iranian population (≥ 91.5 cm for men, ≥ 85.5 cm for women) whereas Nilsson et al. [18] used different cut-offs (WC ≥ 94 cm for men, ≥ 80 cm for women). Another explanation may be the size of waist circumference. Median of waist circumference in males was greater than that of women in the Swedish study [18]. On the contrary, in our study men and women did not differ in terms of waist circumference. This discrepancy could be attributed to the difference in body composition and distribution of fat. Epidemiological studies have shown the difference in the distribution of fat between South Asians and Whites (Caucasians) [30-32].

In the current study, OWC had the ability to discriminate among IR and non-IR males. Parity and menopause may justify this difference. Findings from the Third National Health and Nutrition Examination Survey (NHANES) III demonstrated that increasing parity was associated with increased WC [33]. Also, CARDIA (Coronary Artery Risk Development In Young Adults) study endorsed NHANES findings [34]. Estrogenic attenuation during menopause contributes to the fat mass augmentation and altered distribution of fat; especially in the abdomen [35-38]. Sowers et al. [39] have reported WC and body fat increase during the menopausal transition [40]. There is controversy whether intra-abdominal fat accumulation is related to the process of ageing or menopause [39,41-44]. Stevens et al. have concluded that studies with an accurate method of body composition measurement ascribed this alteration to menopause rather than age [45].

The results of this study provide a very simple screening method for assessment of insulin resistance in older people especially older men and for the aged people who are at higher risk, more evaluation is carried out.

Some limitations of this study deserve comment. Due to the cross sectional nature of this study, we were unable to estimate relative risk of being IR for those above and below the optimal WC cutoff. In addition, the limited number of participants makes generalization of our findings difficult in clinical practice in the elderly.

Also we did not collect the data about physical activity of the participants. The physical activity could confound the reported relationship. However the data of health monitoring of KCF did not show a difference between all the residents in term of the physical activity.

## Conclusion

The fasting blood sugar measure is required to identify IR in elderly Iranian women. In elderly Iranian men, WC seems to perform as well as IDF criteria at predicting IR. Also measured TG may be useful for diagnosis of metabolic syndrome among older Iranian men. We suggest further studies be carried out with larger samples of elderly subjects to determine optimal WC cutoff in elderly IR subjects.

## Competing interest

The authors declare that they have no competing interest.

## Authors’ contributions

MM, MSc; She has participated in designing, conducting, and she was the main author in writing of the manuscript. She read and approved the final manuscript. FS, MD, MPH; He was the main designer of the study and has participated in conducting and writing the manuscript also, he has analyzed the study. He read and approved the final manuscript. HF, MD; He was the principle investigator of the study and finally he read and approved the manuscript. MRA, He had main participation in design, carry out and writing the manuscript. He read and approved the final manuscript. MG, MSc, She has participated in the collection of the data and in writing of the manuscript. She is unfortunately died and could not read the final revised the manuscript. NZS, MSc; She has contributed in the collection of the data. She read and approved the final manuscript. ZB, BSc; She has contributed in the collection of the data. She read and approved the final manuscript. YT, MD; He has contributed in analyzing and writing the manuscript. He read and approved the final manuscript. NN, BSc; She was executive manager of the study and also, has participated in writing the manuscript. She read and approved the final manuscript. BL MD; He was advised on the study and approved the manuscript finally. He read and approved the final manuscript. All authors reads and approved the final manuscript.
